# How Media Stories in Low- and Middle-Income Countries Discussed the U.S. Food and Drug Administration’s Modified Risk Tobacco Product Order for IQOS

**DOI:** 10.1093/ntr/ntad092

**Published:** 2023-06-13

**Authors:** Meagan O Robichaud, Tyler Puryear, Joanna E Cohen, Ryan David Kennedy

**Affiliations:** Institute for Global Tobacco Control, Department of Health, Behavior and Society, Johns Hopkins Bloomberg School of Public Health, Baltimore, MD, USA; Institute for Global Tobacco Control, Department of Health, Behavior and Society, Johns Hopkins Bloomberg School of Public Health, Baltimore, MD, USA; Institute for Global Tobacco Control, Department of Health, Behavior and Society, Johns Hopkins Bloomberg School of Public Health, Baltimore, MD, USA; Institute for Global Tobacco Control, Department of Health, Behavior and Society, Johns Hopkins Bloomberg School of Public Health, Baltimore, MD, USA

## Abstract

**Introduction:**

In 2020, the U.S. Food and Drug Administration authorized the marketing of IQOS as a modified risk tobacco product (MRTP) with reduced exposure information (reduces exposure to harmful chemicals compared to cigarettes) but prohibited Philip Morris International from making reduced risk claims (reduces risk of disease compared to cigarettes). We aimed to assess how news media in low- and middle-income countries (LMICs) discussed this authorization and whether articles discussed IQOS as a reduced exposure versus reduced risk product.

**Aims and Methods:**

News articles published between July 7, 2020 and January 7, 2021 were obtained by searching Tobacco Watcher (www.tobaccowatcher.org), a surveillance platform for tobacco-related news. Articles were eligible if they were published in an LMIC and mentioned the IQOS MRTP order. Non-English language articles were professionally translated. Articles were double coded to identify country of origin, reduced risk and reduced exposure language, discussions of potential impacts of the authorization on regulations in LMICs, and quotes from tobacco industry and public health stakeholders.

**Results:**

We identified 50 eligible articles published in 20 LMICs. Twenty-six (52%) and 40 (80%) included reduced risk and reduced exposure language, respectively. Twenty-two (44%) discussed potential impacts of the MRTP order on regulations in LMICs. Thirty (60%) included quotes from tobacco industry representatives, 6 (12%) included quotes from public health or medical professionals, and 2 (4%) included both.

**Conclusions:**

News articles in LMICs frequently misreported the MRTP order by using reduced risk language. The authorization is potentially being used to shape perspectives on tobacco regulations in LMICs. Tobacco control experts need to more frequently share their perspectives with the news media.

**Implications:**

News articles from LMICs frequently misrepresented the IQOS MRTP order by using reduced risk language (reduces harm compared to cigarettes) rather than only using reduced exposure language (reduces exposure to harmful chemicals compared to cigarettes). Many articles referred to IQOS as a “better alternative” to cigarettes without specifically referencing reduced risk. Few articles included perspectives from public health or medical professionals, while most included tobacco industry quotes, suggesting that tobacco control experts need to more frequently engage with the news media. These findings also highlight how the U.S. FDA’s actions can potentially shape perspectives on tobacco product regulations in LMICs.

## INTRODUCTION

Heated Tobacco Products (HTPs) are often presented as a safer alternative to combustible cigarettes.^1–3^ The global HTP market was valued at more than USD $28.7 billion in 2021—compared to USD $1.4 billion in 2016—and is projected to grow to USD $52.8 billion by 2025.^4^ IQOS, Philip Morris International’s (PMI’s) HTP, was launched in 2014 and was available in 68 markets globally as of August 2022—compared to 25 markets for glo, British American Tobacco’s HTP—and had been launched in 16 low- and middle-income countries (LMICs) by 2021.^5,6^ In 2021, IQOS held 61% of the global HTP device market, and the HEETS tobacco sticks that are inserted into IQOS devices comprised more than 70% of the market for HTP sticks.^6^

Euromonitor International data for 21 LMICs shows that HTP use remains low in most countries.^7^ Less than 1% of adults use HTPs in 13 out of 21 countries, and only Kazakhstan has a use prevalence above 5% (6.8% of adults).^7^ LMICs in the WHO European Region showed the most growth in HTP use, particularly in Russia, Ukraine, and Kazakhstan, where use prevalence grew by 2.5 million, 446 000, and 344 000 users since 2019, respectively.^7^ In South Africa, the retail value increased from USD $32.8 million to USD $224.4 million between 2017 and 2021, but adult use prevalence was below 0.1% in 2021.^7,8^ The market in Latin America grew relatively little (USD $2.2 million in 2017 to USD $26.1 million in 2021), even in LMICs where IQOS was officially launched (Colombia, Dominican Republic, and Guatemala).^7,8^

Independent, epidemiological research into the short- and long-term health effects of HTPs is limited. One study found that current HTP use was associated with persistent respiratory symptoms among youth never and former smokers.^9^ A systematic review of in vitro and human studies suggested that HTP use may negatively impact lung function and increase susceptibility to respiratory diseases.^10^ HTPs also contain nicotine, which can harm brain development and fetal development.^11^ The effectiveness of HTPs for smoking cessation remains unclear, and dual use of cigarettes and HTPs is common among individuals who use HTPs, raising questions about potential population health benefits of HTPs.^12,13^ While studies have demonstrated that HTPs expose users to lower levels of several harmful and potentially harmful chemicals (HPHCs) compared to cigarette smoke, it is unclear if this reduced exposure is sufficient to reduce the risk of tobacco-related disease, given variation in health effects and potencies of HPHCs.^14,15^ IQOS aerosol also has higher levels of 56 other substances compared to cigarette smoke, and information about the toxicity of these constituents is limited.^14^ Thus, reduced exposure to certain HPHCs in IQOS may not translate into reduced risk of disease.^15^

This distinction between reduced exposure and reduced risk is reflected in Modified Risk Tobacco Product (MRTP) authorizations from the U.S. Food and Drug Administration (FDA), which allow tobacco manufacturers to claim that switching from conventional cigarettes to their product reduces exposure to certain substances or reduces users’ risk of disease. A *risk* modification order is issued if the manufacturer demonstrates in their MRTP application that a product will “significantly reduce harm and the risk of tobacco-related disease” and “that the product, as actually used by consumers, *will* benefit the health of the population as a whole,” accounting for both people who use and do not use tobacco.^16,17^ If there is insufficient evidence to support issuing a risk modification order, and such evidence cannot be obtained without long-term epidemiological studies, the FDA may issue an *exposure* modification order, permitting companies to market their product as “containing a reduced level of or presenting a reduced exposure to a substance or as being free of a substance when the issuance of the order is *expected to* benefit the health of the population.”^16,17^ Exposure modification orders are issued if evidence suggests that “substantial reduction in morbidity and mortality among individual tobacco users is *reasonably likely* in subsequent studies.”^16,17^

On July 7, 2020, the U.S. FDA authorized the marketing of IQOS as an MRTP with reduced exposure information, allowing PMI to make the following claims when marketing IQOS in the United States:

“AVAILABLE EVIDENCE TO DATE:

- The IQOS system heats tobacco but does not burn it.
- This significantly reduces the production of harmful and potentially harmful chemicals.
- Scientific studies have shown that switching completely from conventional cigarettes to the IQOS system significantly reduces your body’s exposure to harmful or potentially harmful chemicals.”^18^

However, since there was insufficient evidence to support issuing a risk modification order for IQOS, PMI is prohibited from marketing IQOS with reduced risk claims, including claims that “switching completely to IQOS presents less risk of harm than continuing to smoke cigarettes” or that switching to IQOS “can reduce the risks of tobacco-related diseases.”^16^

Tobacco control stakeholders have found several examples of news articles, including in Mexico, Vietnam, Zimbabwe, and other LMICs, misrepresenting the MRTP order by stating that the FDA found IQOS to be less harmful or a reduced risk product.^19–22^ Additionally, studies suggest that PMI has used the FDA authorization as an opportunity to advocate for “risk based” regulations or to encourage countries to reconsider bans on HTPs.^19,20,22^ Given the importance of the news media in shaping discourse on tobacco control issues, news articles discussing the IQOS MRTP order are an important channel of communication to study. However, it is unclear approximately what proportion of these news articles include such misrepresentations. The extent to which public health perspectives versus tobacco industry perspectives are included in news media articles about the MRTP order is also unclear.

This study will assess how the FDA’s MRTP order for IQOS was discussed in the news media in LMICs. Specifically, this study aims to: (1) Identify which LMICs have published news articles covering the IQOS MRTP order; (2) Determine if the IQOS authorization is correctly discussed as an exposure modification order or IQOS is discussed as a reduced exposure product (reduced exposure language) rather than a risk modification order or reduced risk product (reduced risk language); (3) Identify perspectives (tobacco industry and public health) included in the articles; and (4) Explore content within news articles suggesting that the FDA’s authorization can or should shape tobacco control policies in LMICs (impact of MRTP order).

## METHODS

### Data Source

News articles reporting the July 7, 2020 MRTP order were obtained by systematically searching Tobacco Watcher (https://tobaccowatcher.globaltobaccocontrol.org/), a surveillance platform that identified and compiled tobacco-related news from 147 countries in 16 languages at the time of data collection.^23^ Tobacco Watcher monitors over 500 000 data sources across all six WHO regions, including sources from 28 out of 47 African Region (AFRO) countries (60%), 28 out of 35 Region of the Americas (AMRO) countries (80%), 16 out of 21 Eastern Mediterranean Region (EMRO) countries (76%), 48 out of 53 European Region (EURO) countries (91%), 9 out of 11 South-East Asian Region (SEARO) countries (82%), and 13 out of 27 Western Pacific Region (WPRO) countries (48%), as well as jurisdictions that are not WHO member states (eg, Hong Kong).^23,24^ Locations covered by Tobacco Watcher are shown in Supplementary Table 1.

Tobacco Watcher includes news articles, blog post, and academic journal articles. Since nearly identical articles are often published in multiple locations, Tobacco Watcher automatically marks highly similar articles as “duplicates.”^24^ Tobacco Watcher also allows for Boolean searching and filtering by several criteria, including location, date, and language.^24^

### Search Strategy and Inclusion Criteria

A list of LMICs, defined as countries classified as Low-Income, Lower-Middle Income, or Upper-Middle Income by the World Bank at the time of data collection (Gross National Income per capita of less than USD $13 205) was obtained from the World Bank.^25^ Articles published within 6 months following the 2020 IQOS MRTP order were identified using Boolean searches (ie, “FDA” or “Food and Drug Administration” + “IQOS” + “country name”) and location search filters in Tobacco Watcher. This study did not assess the reach of content related to the MRTP order and instead aimed to characterize unique articles. Therefore, articles marked as duplicates by Tobacco Watcher were excluded. This study included articles from news outlets, defined as media websites providing updates on current events in multiple topic areas (eg, politics, health, entertainment) or a focused topic area (eg, stocks, health). Articles were eligible if they mentioned the July 7, 2020 FDA authorization for IQOS, were published between July 7, 2020 and January 7, 2021, and were published in an LMIC. Searches were restricted to the specified time frame, and the publication date in each article was noted to ensure this criterion was met. Publishing location was determined by Country Code Top-Level Domains in URLs (eg, “.ph” for the Philippines), or addresses and telephone numbers listed on news outlets’ “Contact” or “About Us” pages. Articles not in English were professionally translated through Language Scientific, Inc.

Articles published in countries classified as High-Income countries by the World Bank, published outside of the specified date range, that were inaccessible (eg, paywall, broken link), or that were academic journal articles or blog posts (ie, specifically labeled as “blogs,” contained tobacco product reviews, or personal websites for a specific person), were excluded.

### Codebook Development and Codes

The codebook (Supplementary Table 2) was informed by FDA guidance distinguishing between risk modification and exposure modification, language used in PMI press releases and annual reports, and studies of news media discourse on tobacco control issues.^17,20,26–28^ Coders were instructed to leave comments during data collection to capture emergent themes and inform codebook refinement.

Codes included article language, publication date, country of publication, and news media outlet section (eg, business, health). The codebook also captured use of the term “MRTP” or “modified risk tobacco product” and presence of reduced risk and reduced exposure language. An article was coded yes for “reduced exposure” if it explicitly stated that IQOS “reduces the production of HPHCs” or substances, “reduces users’ exposure to HPHCs” or substances, or contains lower levels of HPHCs; if it described IQOS as a “modified exposure product”; or mentioned “exposure modification orders” from the FDA. An article was coded yes for “reduced risk” if it described IQOS as “less harmful,” a “reduced risk” product, or a “safer alternative”; stated that the FDA described IQOS as a reduced risk, safer, or less harmful product or that the FDA endorsed IQOS as a harm reduction product; or suggested that IQOS is one type of “less harmful,” “alternative,” or safer product (eg, alongside e-cigarettes). The codebook also captures quotes from tobacco industry employees and public health or medical professionals, content discussing impact of the MRTP order (eg, suggesting that the FDA authorization should inform tobacco product regulations elsewhere), and language related to PMI’s efforts to decrease combustible tobacco use.

### Data Analysis and Synthesis

Each article was independently double-coded using REDCap, a secure web-based data collection platform.^29,30^ The level of agreement between coders varied between codes, and variables with at least 60% agreement were included in the analysis (percent agreement range: 60%–98%; Krippendorf’s α range: −0.09 to 0.86). Coders met to discuss and resolve discrepancies in coding and to discuss emerging themes, and a third member of the study team resolved remaining disagreements. A final “merged” code with the agreed upon codes were used in the results.

Article publishing locations were recoded into WHO regions (AFRO, AMRO, EMRO, EURO, SEARO, or WPRO) for the analysis. The number of articles for each code was determined, and exemplary quotes for each code were identified.

## RESULTS

### LMICs Covering the IQOS MRTP Order

The search yielded 260 results. Of these, 168 duplicates (ie, articles that came up in more than one search) were excluded, leaving 92 unique articles. Upon review, 42 were excluded because they were not published in an LMIC (26), did not mention the 2020 IQOS MRTP order (13), were inaccessible (2), or were not news articles (1), leaving 50 eligible articles in the analysis.

Article characteristics are described in Table 1. Articles were published in 20 different LMICs, with 15 (30%) in WPRO, 14 (28%) in AFRO, 13 (26%) in AMRO, 4 (8%) in SEARO, 3 (6%) in EMRO, and 1 (2%) in EURO. Forty-eight percent of articles were in English, 26% in Spanish, 8% in Mandarin, 8% in Vietnamese, 4% in Arabic, 4% in Bahasa Indonesia, and 2% in French. Nearly half (46%) of the articles were published in sections pertaining to business, economy, or finance. Twelve percent of articles were published in a health-related section. The remaining articles were published as opinion pieces (4%), in other sections (20%), or the section was unclear (18%).

**Table 1.** Characteristics of Digital News Articles Published in LMICs (July 7, 2020 Through January 7, 2021)

	Number of articles (%) *N* = 50
WHO region (countries)	
Western Pacific Region (China, Malaysia, the Philippines, and Vietnam)	15 (30%)
African Region (Kenya, Malawi, Nigeria, Senegal, and Zambia*)	14 (28%)
Region of the Americas (Argentina, Colombia, Costa Rica, Dominican Republic, Guatemala, and Mexico)	13 (26%)
South-East Asia Region (India and Indonesia)	4 (8%)
Eastern Mediterranean Region (Egypt, West Bank and Gaza)	3 (6%)
European Region (Moldova)	1 (2%)
Language	
English^†^	24 (48%)
Spanish	13 (26%)
Mandarin	4 (8%)
Vietnamese	4 (8%)
Arabic	2 (4%)
Bahasa Indonesia	2 (4%)
French	1 (2%)
Newspaper section	
Business, Finance, or Economy	23 (46%)
Health	6 (12%)
Opinion	2 (4%)
Other^**^	10 (20%)
Unclear	9 (18%)
Article publication date	
July 7 through July 31, 2020	18 (36%)
August 2020	12 (24%)
September 2020	6 (12%)
October 2020	4 (8%)
November 2020	4 (8%)
December 2020	5 (10%)
January 1 through January 7, 2021	1 (2%)

^*^One article was published from a news source that has offices in multiple locations, primarily in Africa.

^†^One article was in English but was likely translated from another language previously.

^**^”Other” includes “National” “News” “Life & Style” “Analysis” “Humanities” “Advertorials” “Bespoke ad” and “Letters.”

### Reduced Exposure and Reduced Risk Language

Table 2 presents the number of articles using reduced risk and reduced exposure language, stratified by use of the term “modified risk tobacco product” or “MRTP.” The majority of articles (*n* = 33, 66%) used the term “modified risk tobacco product” or “MRTP.” Most articles (*n* = 26, 52%) included reduced risk language and therefore did not accurately represent the MRTP authorization as an exposure modification order. Five articles (10%) included only reduced risk language, while 21 (42%) included both reduced risk and reduced exposure language. Nineteen articles (38%) included only reduced exposure language. Three articles (6%) used the term “MRTP” but did not include reduced exposure or reduced risk language. Three additional articles (6%) used the term “MRTP” but included reduced risk language only. One emergent theme identified in 22 articles (44%) was language describing IQOS as a “better alternative” or similar language (eg, better choice), which frequently appeared in tobacco industry quotes.

**Table 2.** Articles Reduced Risk and Reduced Exposure Language (Stratified by Use of Term “MRTP” or “Modified Risk Tobacco Product”)

	Number of articles (%)	Example
“MRTP” or “Modified Risk Tobacco Product”	33 (66%)	
Reduced Risk Language Only	3 (6%)	“On July 8, the U.S. FDA officially announced its approval of the **MRTP (modified risk tobacco product)** application for Philip Morris’ IQOS… IQOS is the world’s first HNB product to **obtain a government’s official harm-reduction endorsement**.” (East Money, China, 07/15/20).
Reduced Exposure AND Reduced Risk Language	15 (30%)	Article titled “U.S. FDA Authorizes Marketing of Heated Tobacco Products: **Less Harmful”** states: “The [U.S. FDA] has officially authorized the marketing of IQOS electrically heated tobacco products in the United States as **Modified Risk Tobacco Products**… they **reduce the chemicals that are harmful to human health** that result from traditional cigarettes.” (El Watan News, Egypt, 07/12/20).
Reduced Exposure Language Only	12 (24%)	Article titled “U.S. **MRTP** Authorization Pathway Explored in PMI’s Latest Scientific Update” states: “…the FDA concluded: ‘Scientific studies have shown that switching completely from conventional cigarettes to the IQOS system significantly **reduces your body’s exposure to harmful or potentially harmful chemicals.**’” (IndiaOnline.in, India, 09/25/2020)
Neither Reduced Exposure nor Reduced Risk Language	3 (6%)	“…the U.S. Food and Drug Administration (FDA) authorized the sale of IQOS as a **modified risk tobacco product (MRTP),** deeming the device a different product and an option for adults who will not stop smoking, Philip Morris explained.” (Investing.com, Mexico, 07/29/2020)
Does NOT Use the Term MRTP^*^	17 (34%)	
Reduced Risk Language Only	2 (4%)	“Specifically, he confirmed the IQOS heated tobacco products receiving FDA’s approval. **The products have fewer health risks than ordinary cigarettes**.” (Investo, Vietnam, 12/21/20)
Both Reduced Exposure AND Reduced Risk Language	6 (12%)	“PMI Vice President Strategic and Scientific Communications Moira Gilchrist claimed that the IQOS product is **low risk** compared to conventional cigarettes… the results of the research show that IQOS **reduces users’ exposure to 15 dangerous chemical substances** to a level approaching smokers who stopped smoking.” (CNN Indonesia, Indonesia, 07/04/2020)
Reduced Exposure Language Only	7 (14%)	“It is worth noting that in early July, the FDA approved the sale of the IQOS tobacco heating system as an **exposure modification order**.” (InfoMarket, Moldova, 07/24/2020)
Neither Reduced Exposure nor Reduced Risk Language	2 (4%)	“...[the U.S. FDA] had denied Philip Morris’ claim that switching from combustible cigarettes to the company’s HTP brand, IQOS, will reduce the risk of disease…” (Premium Times, Nigeria, 07/29/20)
Better Alternative Language	22 (44%)	“‘Today’s decision makes it possible to inform these adults that switching completely to IQOS is a **better option** than continuing to smoke,’ Calantzopoulos said.” (pulzo, Colombia, 07/10/20)

^*^Three articles used term “modified risk product,” rather than “modified risk tobacco product.”

### Perspectives Included in Articles

Thirty-eight articles (76%) included quotes from either the tobacco industry or public health or medical professionals (Table 3). Thirty articles (60%) included quotes from a tobacco industry employee and no quotes from public health stakeholders. Tobacco industry quotes frequently discussed the need to regulate non-combusted tobacco products, including IQOS, “differently” from cigarettes, PMI’s investment in research and development for noncombustible products, and the need for regulations to encourage innovation in this area. Some PMI quotes also discussed the company’s efforts to prevent youth access to IQOS, referencing what PMI calls “Good Conversion Practice” (ie, age verification, confirming if prospective buyers smoke before discussing the “benefits of switching” to IQOS).

**Table 3.** Quotes Included in Articles

	Number of articles (%)	Examples
**Tobacco Industry Representative Only**	30 (60%)	“The FDA undertook a scientific and regulatory evaluation of IQOS and determined that it is ‘appropriate for the promotion of public health’ said **Dr. Gizelle Baker, director of Global Scientific Engagement at PMI.”** (India Online, India, 09/26/2020) “We see smoke-free products as an opportunity to reduce the smoking incidence in the Philippines for the benefit of the public health and society at large,” **PMFTC [affiliate of Philip Morris International] President Denis Gorkun** said in a statement. (Inquirer.net, Philippines, 12/16/20)
**Public Health or Medical Professional Only**	6 (12%)	“‘ENDS—the name for e-cigarettes or heated tobacco products—have not been established as methods for cessation, and recent research has established harm to health,’ said **Nydia Amador, president of RENATA [National Anti Tobacco Network of Costa Rica].”** (am Prensa, Costa Rica, 07/27/20) “In an interview with **Victor Mithi, President of the Medical Society of Malawi**, he was of the view that smokers should be encouraged to quit as opposed to seeking less harmful options. ‘We do not have enough local scientific data on the impacts of tobacco smoking but we would be more inclined to encourage smokers to quit smoking completely instead of opting for other tobacco products,’ said Mithi.” (Face of Malawi, Malawi, 12/07/20)
**Both**	2 (4%)	“‘This is another step forward. Many developing countries look to the US FDA for guidance, not just in alternative tobacco products but in medicines and vaccines,’ he [**Professor Tikki Pang, visiting professor at the Yong Loo Lin School of Medicine at the National University of Singapore**] added… **Dr Moira Gilchrist, the vice-president of Strategic and Scientific Communications at Philip Morris International (PMI)** said that even with this authorization that the FDA continually monitors this status. ‘There is continual oversight, in order to remain on the market and in order to continue to bring these messages to consumers, we have to show that the product is appropriate to promote the public health for its entire life cycle,’ [s]he said.” (malaysiakini, Malaysia, 11/06/20) “‘Data submitted by PMI show that commercializing these products with the above approved information can help adult smokers who are addicted to smoking lit cigarettes transition away from it, thus minimize the body’s exposure to harmful chemicals, but on condition that they change completely (over to IQOS),’ said **Mitch Zeller, J.D.**...In a statement, **Philip Morris CEO Andre Calantzopoulos** praised the FDA’s decision and said it showed that ‘IQOS is a tobacco product fundamentally different from cigarettes, and is a better choice for adults who choose to continue smoking.’ **Altria CEO Billy Gifford** called the decision ‘an important step forward’ for adult smokers, judging that the FDA’s approval allows the company to provide information about the benefits of switching to IQOS product.” (LAO ĐỘNG, Vietnam, 07/09/2020)

Six articles (12%) included quotes from only a public health or medical professional (and no tobacco industry quotes), and two (4%) included quotes from both the tobacco industry and public health or medical professionals. Four of these articles quoted then Director of the FDA Center for Tobacco Products, Mitch Zeller, in which he explained the exposure modification order, emphasizing that individuals must completely switch from cigarettes to IQOS to reduce their exposure to HPHCs. Other health stakeholders quoted included physicians, public health researchers, leaders of medical societies, or employees of health-oriented non-governmental organizations (eg, President of the National Anti-Tobacco Network of Costa Rica). Two public health stakeholders expressed favorable attitudes toward IQOS and other noncombustible products as potential “harm reduction” strategies for people who smoke, while three expressed skepticism or mixed views.

### Impact of MRTP Order

Quotes discussing potential impacts of the MRTP order (Table 4) were found in articles from countries with little to no clear regulations for HTPs (Argentina, Dominican Republic, Guatemala, Kenya, Nigeria, Vietnam, and Zambia) as well as in countries that regulate them as tobacco products (Costa Rica and Malaysia) or as separate product categories (Egypt, Indonesia, Moldova, the Philippines, and Senegal).^31^ Twenty-two articles (44%) discussed how the FDA authorization should, or should not, influence tobacco product regulations outside of the United States, including calls for HTPs to be regulated “differently” based on the “degrees of harmfulness” of various tobacco products (ie, more stringent regulation for cigarettes, less stringent regulations for noncombustible products). Four articles published in countries where HTPs are officially banned (India and Mexico) suggested that these bans should be reconsidered in light of the FDA authorization (eg, article titled “Philip Morris Asks Government for Talks on IQOS Ban”). Other articles suggested that the FDA authorization can serve as an example for tobacco product regulations elsewhere, as exemplified in the following quote from Stacey Kennedy (then President, South, and South East Asia at PMI): “Use that [U.S. FDA decision] as a starting block rather than starting all the way from square one, and I think that’s really a critical way that countries can accelerate by looking at the transparency and processes of other countries.”

**Table 4.** Impact of FDA MRTP Order

	**Number of articles (%)**	**Examples**
**Potential Impact of FDA Authorization on Regulations in Other Countries**	22 (44%)	“IQOS products are fundamentally different from traditional cigarettes; therefore, **they need to be regulated differently, as stated by FDA**. Now, more urgently than ever, there is the need for a fundamentally different dialogue about a collaborative approach towards a smoke-free future.” (Công Thương, Vietnam, 08/01/20) “‘Last year, the excise tax on IQOS was increased 11 times (despite the fact that electronic cigarettes are still not subject to excise tax)! And in this we see a great injustice, because the innovative product was equated to a regular pack of cigarettes. **We are confident that tax policy should take into account the risks of products and tie the excise tax exclusively to the degree of their harmfulness**,’ Naumenko stressed... It is worth noting that in early July, the FDA approved the sale of the IQOS tobacco heating system as an exposure modification order.” (InfoMarket, Moldova, 07/24/20)
**Economic Impact of the FDA Authorization**	12 (24%)	“With rising numbers of smokers in sub-Saharan Africa, concerns regarding risk exposure to tobacco have been well documented. The impact on the entire industry, from farmers to consumers has always been a source of concern: **who will support the farmers earning a living through their trade? This could change after a landmark decision in the industry.**” (Mwebantu, Zambia, 08/07/20) “…the **rise in global demand for HNB [heat not burn] products will help new tobacco business cash in**, in terms of business revenue performance.” (36kr, China, 08/25/20)
**Accessibility to People in LMICs**	5 (10%)	“Calantzopoulos believes that the US FDA’s authorization would help PMI further accelerate the transition of American adults away from cigarettes. Will the same thing happen in the Philippines? **I can’t imagine Filipinos in poorer communities shifting to IQOS, which is a pricey device**.” (Philstar Global, Philippines, 7/23/20) “…experts affirm that the African consumer has **little exposure to knowledge of safer nicotine and tobacco products…**” (Upshot Reports, Nigeria, 08/07/20)

In contrast, some articles suggested that the FDA authorization should *not* impact regulations in their respective country. One article from Costa Rica stated, “The National Anti-Tobacco Network of Costa Rica (RENATA) and a number of organizations spoke out and warned that the [IQOS MRTP] authorization… opens a dangerous door that puts children and young people at risk.” Similarly, an article from Mexico included a statement from two government health agencies expressing concerns about the FDA’s authorization being used to mislead lawmakers and the public, stating that the MRTP order does not have authority outside of the United States and that IQOS is not considered a reduced risk product or a smoking cessation tool under Mexican government bodies.

One emergent theme identified in 12 articles (24%) was the potential economic impact of the IQOS MRTP order, including improved sales for tobacco growers and impact on tobacco company stocks or tobacco businesses more generally. For example, one article suggested that “the smokeless cigarette innovation [IQOS] also bring[s] a new ray of hope to the African tobacco industry in which PMI buys its tobacco from, including Malawi—whose earnings from the green gold has sharply declined in recent years.” Another emergent theme, discussed in five articles (10%), related to accessibility of electronic tobacco and nicotine products (including IQOS) to people in LMICs. Reasons for limited accessibility highlighted in these articles were affordability of IQOS (ie, higher cost relative to cigarettes), regulations limiting access to these products, and low awareness of non-combusted products.

### Additional Article Themes

Fifty-six percent of articles referenced PMI’s efforts to “create a smoke-free world” (ie, “replacing” combustible tobacco with noncombustible products, such as IQOS). For example, according to an article from Nigeria, a PMI executive stated that, “PMI remains totally committed to ensuring that smoke-free products which are safer, replace the current cigarettes as soon as possible.” This theme also appeared in some article titles (eg, “Philip Morris Declares War on Tobacco Smoke”).

Additionally, two articles indicated that the article was sponsored or promoted, with one article from Guatemala tagged as “sponsored content” and another from Mexico indicating “branded content.”

## DISCUSSION

While 38% of the articles correctly describe the MRTP order by using reduced exposure language only, half incorrectly report this authorization by stating that the FDA has determined IQOS to be a reduced risk product or that IQOS is a reduced harm product, suggesting that reduced exposure is sometimes misreported as reduced risk in the news media. This is consistent with findings from independent experimental studies and studies of consumer understanding submitted by PMI as part of their MRTP application, which found that consumers who viewed reduced exposure claims reported lower risk perceptions.^16,32^ News articles frequently contained language describing IQOS or other noncombustible products as “better alternatives” for smokers. Such language was often present in tobacco industry quotes and was in PMI’s press release discussing the MRTP order for IQOS.^33^ While not directly referring to IQOS as a reduced risk product, such language can potentially add to confusion surrounding reduced risk versus reduced exposure. Additionally, 44% of articles discussed the potential impact of the FDA’s authorization on regulations outside of the United States, such as by stating that the FDA’s authorization should be looked to as an example for other countries’ tobacco product regulations, suggesting that the influence of the U.S. FDA’s decisions may reach beyond the United States, including into LMICs.

While articles discussing the MRTP order for IQOS were found in all six WHO regions, 74% of articles were published in AFRO, AMRO, and WPRO LMICs. It is unclear why the search yielded few articles in EURO LMICs, where IQOS is more widely available and for which Tobacco Watcher coverage is highest, but several articles in AFRO and WPRO LMICs, where IQOS is less available. PMI previously identified the Philippines and Vietnam as “key markets” and launched BONDS by IQOS in the Philippines in 2022, suggesting that the company had plans at the time of data collection to expand the market for IQOS in WPRO LMICs.^34^

Several articles discussed PMI’s efforts to replace combusted tobacco with IQOS and other non-combusted tobacco products. It is possible that PMI used the press coverage of the FDA’s MRTP authorization as a public relations (PR) and marketing opportunity, which continued months after the MRTP order. While public health and tobacco control organizations, including the Campaign for Tobacco Free Kids and the World Health Organization, also released statements discussing the MRTP authorization for IQOS, our findings suggest that these perspectives received little press coverage in LMICs compared to PMI’s press releases and quotes from PMI leadership.^35,36^

Emergent themes highlighted issues related to the MRTP order that may be particularly salient to LMICs, including limited access to IQOS due to its high cost relative to cigarettes or because of current regulations. Some articles also discussed the potential for the FDA’s authorization to increase tobacco sales, thus benefitting tobacco farmers, which may be salient to several LMICs since LMICs are the primary source of tobacco leaves and because the tobacco industry has argued that stringent tobacco control measures will harm tobacco farmers.^37,38^

### Strengths and Limitations

This article has several strengths, such as the inclusion of non-English language articles and the professional translation of these articles. This study’s in-depth content analysis of news media articles in LMICs adds to prior research on this topic by providing detailed examples of language used to discuss the MRTP order (both accurate and inaccurate), providing an estimate of how widespread misrepresentations of the authorization may be in the news media in LMICs, and by assessing the extent to which public health and tobacco industry perspectives were included in articles discussing the MRTP order.

This study also has several limitations. First, the news articles obtained from Tobacco Watcher may not be a complete compilation of news articles from LMICs on the IQOS MRTP authorization, since Tobacco Watcher only includes digital media stories and does not include print-only news outlets. Additionally, at the time of data collection, articles in languages from key LMICs, including Pakistan (Urdu) and the Philippines (Filipino), were not available. Second, this study could not assess the reach of the news media articles analyzed because information about the number of times an article was viewed or shared was limited. Finally, the small sample size did not allow for any statistical analyses to compare article characteristics by WHO region, date, or language.

### Recommendations

These findings have several implications for future tobacco control research and for communicating about tobacco regulations. Future studies can assess how media coverage, including the news media and social media, shape consumer perspectives about IQOS, other noncombustible products, and regulations for these products, both in the United States and in LMICs. In light of our findings suggesting that the FDA’s MRTP authorization can potentially shape discussions surrounding tobacco product regulations, there is a need for continued surveillance of regulations for HTPs and other noncombustible tobacco products, and continued monitoring of tobacco industry activities and interference tactics in LMICs, including direct contact with lawmakers and governing bodies, arguments that regulations like the MRTP authorization will benefit tobacco farmers, and positive PR coverage of the tobacco industry (eg, claims about creating a “smoke-free future”).

This study also highlights a lack balance in the perspectives included in stories about tobacco control issues, with most articles in the study including quotes only from the tobacco industry and relatively few quoting public health stakeholders. Journalism researchers have raised concerns about overreliance on company press releases when writing news stories, which frequently lack information about wider contextual factors and generally paint companies in a positive light.^39^ These concerns are relevant for tobacco company press releases and statements from tobacco company employees, particularly when news media outlets are under-resourced. Tobacco control stakeholders can improve journalistic balance in news stories by more effectively distributing and promoting their own press releases.

## Supplementary Material

A Contributorship Form detailing each author’s specific involvement with this content, as well as any supplementary data, are available online at https://academic.oup.com/ntr.

ntad092_suppl_Supplementary_Tables

## Data Availability

The data underlying this article will be shared on reasonable request to the corresponding author.
