# Supplementary material for: How Media Stories in Low- and Middle-Income Countries Discussed the U.S. Food and Drug Administration’s Modified Risk Tobacco Product Order for IQOS
Source: Nicotine Tob Res. 2023 Jun 13;25(10):1659–66. doi: 10.1093/ntr/ntad092 (PMC10445252; doi:10.1093/ntr/ntad092)
Supplement: ntad092_suppl_Supplementary_Tables [file ntad092_suppl_supplementary_tables.docx]

**Supplementary Table 1: Countries Covered by Tobacco Watcher (by WHO Region)**

| **COUNTRY OR JURISDICTION** | **WORLD BANK INCOME CLASSIFICATION (January 2021)** |
| --- | --- |
| **African Region (AFRO)** | |
| ***Algeria*** | Lower-middle-income |
| ***Angola*** | Lower-middle-income |
| ***Botswana*** | Upper-middle-income |
| ***Cameroon*** | Lower-middle-income |
| ***Congo, Dem. Rep*** | Low-income |
| ***Eritrea*** | Low-income |
| ***Eswatini*** | Lower-middle-income |
| ***Ethiopia*** | Low-income |
| ***The Gambia*** | Low-income |
| ***Ghana*** | Lower-middle-income |
| ***Kenya*** | Lower-middle-income |
| ***Lesotho*** | Lower-middle-income |
| ***Liberia*** | Low-income |
| ***Madagascar*** | Low-income |
| ***Malawi*** | Low-income |
| ***Mozambique*** | Low-income |
| ***Namibia*** | Upper-middle-income |
| ***Nigeria*** | Lower-middle-income |
| ***Rwanda*** | Low-income |
| ***Senegal*** | Lower-middle-income |
| ***Sierra Leone*** | Low-income |
| ***Somalia*** | Low-income |
| ***South Africa*** | Upper-middle-income |
| ***Sudan*** | Low-income |
| ***Tanzania*** | Lower-middle-income |
| ***Uganda*** | Low-income |
| ***Zambia*** | Low-income |
| ***Zimbabwe*** | Lower-middle-income |
| **Region of the Americas (AMRO)** |  |
| ***Argentina*** | Upper-middle-income |
| *Bahamas, The* | High-income |
| ***Belize*** | Upper-middle-income |
| ***Brazil*** | Upper-middle-income |
| *Canada* | High-income |
| *Chile* | High-income |
| ***Colombia*** | Upper-middle-income |
| ***Costa Rica*** | Upper-middle-income |
| ***Cuba*** | Upper-middle-income |
| ***Dominica*** | Upper-middle-income |
| ***Dominican Republic*** | Upper-middle-income |
| ***Ecuador*** | Upper-middle-income |
| ***El Salvador*** | Lower-middle-income |
| ***Grenada*** | Upper-middle-income |
| ***Guatemala*** | Upper-middle-income |
| ***Guyana*** | Upper-middle-income |
| ***Honduras*** | Lower-middle-income |
| ***Jamaica*** | Upper-middle-income |
| ***Mexico*** | Upper-middle-income |
| ***Nicaragua*** | Lower-middle-income |
| *Panama* | High-income |
| ***Paraguay*** | Upper-middle-income |
| ***Peru*** | Upper-middle-income |
| ***Suriname*** | Upper-middle-income |
| *Trinidad and Tobago* | High-income |
| *United States* | High-income |
| *Uruguay* | High-income |
| ***Venezuela, RB^*^*** | Upper-middle-income |
| **Eastern Mediterranean Region (EMRO)** |  |
| ***Afghanistan*** | Low-income |
| *Bahrain* | High-income |
| ***Egypt, Arab Rep.*** | Lower-middle-income |
| ***Iran, Islamic Rep.*** | Lower-middle-income |
| ***Iraq*** | Upper-middle-income |
| ***Jordan*** | Upper-middle-income |
| *Kuwait* | High-income |
| ***Lebanon*** | Lower-middle-income |
| ***Morocco*** | Lower-middle-income |
| *Oman* | High-income |
| ***Pakistan*** | Lower-middle-income |
| *Qatar* | High-income |
| *Saudi Arabia* | High-income |
| ***Syrian Arab Republic*** | Low-income |
| ***Tunisia*** | Lower-middle-income |
| *United Arab Emirates* | High-income |
| **European Region (EURO)** |  |
| *Andorra* | High-income |
| ***Armenia*** | Upper-middle-income |
| *Austria* | High-income |
| ***Azerbaijan*** | Upper-middle-income |
| *Belgium* | High-income |
| ***Belarus*** | Upper-middle-income |
| ***Bulgaria*** | Upper-middle-income |
| *Croatia* | High-income |
| *Cyprus* | High-income |
| *Czech Republic* | High-income |
| *Denmark* | High-income |
| *Estonia* | High-income |
| *Finland* | High-income |
| *France* | High-income |
| *Germany* | High-income |
| *Greece* | High-income |
| *Hungary* | High-income |
| *Iceland* | High-income |
| *Ireland* | High-income |
| *Israel* | High-income |
| *Italy* | High-income |
| ***Kazakhstan*** | Upper-middle-income |
| ***Kyrgyz Republic*** | Lower-middle-income |
| *Latvia* | High-income |
| *Lithuania* | High-income |
| *Luxembourg* | High-income |
| *Malta* | High-income |
| ***Moldova*** | Upper-middle-income |
| ***Montenegro*** | Upper-middle-income |
| *Netherlands* | High-income |
| ***North Macedonia*** | Upper-middle-income |
| *Norway* | High-income |
| *Poland* | High-income |
| *Portugal* | High-income |
| ***Romania^†^*** | Upper-middle-income |
| ***Russian Federation*** | Upper-middle-income |
| ***Serbia*** | Upper-middle-income |
| *Slovak Republic* | High-income |
| *Slovenia* | High-income |
| *Spain* | High-income |
| *Sweden* | High-income |
| *Switzerland* | High-income |
| ***Tajikistan*** | Lower-middle-income |
| ***Turkey*** | Upper-middle-income |
| ***Turkmenistan*** | Upper-middle-income |
| ***Ukraine*** | Lower-middle-income |
| *United Kingdom* | High-income |
| ***Uzbekistan*** | Lower-middle-income |
| **South East Asia Region (SEARO)** |  |
| ***Bangladesh*** | Lower-middle-income |
| ***Bhutan*** | Lower-middle-income |
| ***India*** | Lower-middle-income |
| ***Indonesia*** | Lower-middle-income |
| ***Korea, Dem. People's Rep.*** | Low-income |
| ***Myanmar*** | Lower-middle-income |
| ***Nepal*** | Lower-middle-income |
| ***Sri Lanka*** | Lower-middle-income |
| ***Thailand*** | Upper-middle-income |
| **Western Pacific Region (WPRO)** |  |
| *Australia* | High-income |
| ***China*** | Upper-middle-income |
| ***Cambodia*** | Lower-middle-income |
| ***Fiji*** | Upper-middle-income |
| *Japan* | High-income |
| *Korea, Rep.* | High-income |
| ***Malaysia*** | Upper-middle-income |
| ***Mongolia*** | Lower-middle-income |
| *New Zealand* | High-income |
| ***Philippines*** | Lower-middle-income |
| ***Samoa*** | Lower-middle-income |
| *Singapore* | High-income |
| ***Vietnam*** | Lower-middle-income |
| **Jurisdictions Without a WHO Region** |  |
| *Hong Kong SAR, China* | High-income |
| *Isle of Man* | High-income |
| ***Global/non-specific*** | N/A |
| *Taiwan* | High-income |
| *Yugoslavia* | N/A (country does not exist) |

***Bold italic font*** indicates the country was included in the searches for this study

*According to the World Bank, “Venezuela has been temporarily unclassified as of July 2021 pending release of revised national accounts statistics” but was classified as upper-middle-income at the time of data collection

†Currently high-income but was classified as upper-middle-income at the time of data collection.

**Supplementary Table 2: Codebook and Description of Codes**

| **CODE** | **DESCRIPTION** |
| --- | --- |
| **ARTICLE CHARACTERISTICS** | |
| **Publishing Location** | Country where the news article is published (countries later recoded as WHO regions).  Determined by Country Code Top-Level Domains in the URL (e.g., “.ph” for the Philippines) or address and/or telephone number listed on the news outlet website’s “contact” or “about us” page. |
| **Publication Date** | Date when the article was published. |
| **Language** | Original language of the article. |
| **News Outlet Section*** | Section of the news outlet (e.g., Health, Business) in which the article was published.  Determined by one of the following:   - Section tagged by the news outlet at the beginning of the article (e.g., “Business” tag linking to Business section written above the article title) - Section indicated in breadcrumb navigation for the article page (e.g., “Home > **Healthcare News** > VIEW: The US FDA Signals a New Path for Smokeless Tobacco”) or the URL (https://www.cnbctv18.com/**healthcare**/view-the-us-fda-signals-a-new-path-for-smokeless-tobacco-6607361.htm) - The focus area of the entire news outlet, as confirmed by “about us” page (e.g., *Investo* from Vietnam focuses entirely on stocks and investing; therefore coded as Other—Stocks) |
| **LANGUAGE DESCRIBING IQOS AND FDA DECISION** | |
| **MRTP** | States that IQOS is an “MRTP” or “Modified Risk Tobacco Product” or otherwise uses the term “MRTP” or “Modified Risk Tobacco Product” to describe IQOS. |
| **Reduced Exposure Language** | Includes language that explicitly references any of the following:   - IQOS “reduces the production of harmful and potentially harmful chemicals” or substances; - IQOS “reduces users’ exposure to harmful and potentially harmful chemicals” or substances; - IQOS is described as a “modified exposure product” - “Exposure modification orders” [from the FDA] - IQOS contains lower levels of harmful and potentially harmful chemicals or substances |
| **Reduced Risk Language** | Includes any of the following language:   - Describes IQOS as “less harmful” [than cigarettes], a “reduced risk” product, or a “safer alternative” [to cigarettes]; - States that the FDA has said that IQOS is a reduced risk, safer, or less harmful product [than cigarettes] or that the FDA has endorsed IQOS as a harm reduction product; - Suggest that IQOS is one of several type of “less harmful,” “alternative,” or safer products (e.g., potentially alongside e-cigarettes and other non-combustible products) |
| **Better Alternative Language*** | Includes language describing IQOS as a “better alternative,” “better choice,” or “better option” than cigarettes, without specifying reduced risk or reduced exposure.  This code is not mutually exclusive from the reduced risk and reduced exposure codes specified above. |
| **PERSPECTIVES INCLUDED IN ARTICLE** | |
| **Tobacco Industry Quote** | Article includes a quote from an employee of Philip Morris International or other tobacco company.  Quotes must be attributed to a specific person within a tobacco control company to be counted (e.g., a quote from a press release that is not attributed to a specific person within Philip Morris does not count). |
| **Public Health or Medical Professional*** | Article includes a quote from at least one of the following:   - A healthcare provider - A representative of a health-oriented government agency (e.g., the US FDA, Ministry of Health) - An individual working in tobacco control - An individual from a health-oriented non-governmental organization (e.g., World Health Organization) - Faculty or researchers from a school of medicine or public health   Quotes must be attributed to a specific person (e.g., a quote from an FDA press release, report, or statement that is not attributed to a specific person does not count). |
| **ADDITIONAL THEMES** | |
| **Creating a “Smoke-Free World”** | Article explicitly mentions Philip Morris International’s claim that the company is “creating a smoke-free world,” or mentions other industry efforts to reduce combusted tobacco use. |
| **Impact of MRTP Order on Regulations Outside of US** | Article suggests that the FDA decision can, should, or should not inform the regulation of HTPs in the respective country (e.g., suggesting that regulations on HTPs should be less stringent based on the MRTP order). |
| **Economic Impact of MRTP Order*** | Article discusses how the MRTP order can impact or has impacted at least one of the following:   - Stocks - Tobacco growing jobs or tobacco growers’ livelihoods - Businesses |
| **Accessibility to Individuals in LMICs*** | Article discusses the extent to which people in LMICs are able to access IQOS, other HTPs, or other non-combustible tobacco products (e.g., e-cigarettes). Access can include availability within specific LMICs as well as the extent to which these products are affordable to people in LMICs. |

*Indicates emergent code
